# Hearts over Time: Cardiovascular Mortality Risk Linked to Long-Term PM_2.5_ Exposure

**DOI:** 10.1289/ehp.120-a205a

**Published:** 2012-05-01

**Authors:** Tanya Tillett

**Affiliations:** Tanya Tillett, MA, of Durham, NC, is a staff writer/editor for *EHP*. She has been on the *EHP* staff since 2000 and has represented the journal at national and international conferences.

Adverse respiratory effects and short-term hospital admissions have been linked to acute exposure to fine particulate matter (PM_2.5_), but few studies have examined mortality risks associated with chronic exposure. Now, in Canada’s first national-level cohort study of the subject, investigators report an association between cardiovascular mortality and long-term exposure to PM_2.5_ [*EHP* 120(5):708–714; Crouse et al.].

Most research examining associations between ambient air pollution and human health has used data generated by ground-based air-pollution monitors over short periods of time. But in the current study, researchers also calculated long-term exposure levels using satellite-based estimates of ground-level PM_2.5_.

Study subjects consisted of 2.1 million adults who were included in the 1991–2001 Canadian census mortality follow-up study. For residents of cities with ground-based air monitors, the investigators calculated average mean annual concentrations of PM_^2.5^_ for the period 1987–2001 and assigned exposure levels to individuals based on their residence during that time. They also calculated exposure estimates for the whole cohort for the period 2001–2006 based on satellite remote sensing observations.

The team linked the census and exposure data to national mortality data to estimate increased risk of death from ischemic heart disease, cerebrovascular disease, cardiovascular disease, circulatory disease, and all nonaccidental causes for each 10-µg/m^3^ increase in PM_^2.5^_. The largest estimated increase in mortality was for ischemic heart disease (31%), followed by deaths from nonaccidental causes, circulatory diseases, and cardiovascular disease (each estimated to increase by 15–16%). In contrast, cerebrovascular disease was not clearly associated with PM_2.5_ exposure.

Strengths of the study include the large sample size and the use of remote sensing satellite data, which allowed exposure estimates to be calculated for subjects living in areas without air-pollution monitors. Limitations include a lack of information on smoking and obesity—two of the primary risk factors for cardiovascular disease—and the inability to account for address changes during the follow-up period. However, with a mean estimated exposure of 8.7 µg/m^3^ (range 1.9–19.2 µg/m^3^), the findings suggest that exposure even to very low amounts of PM_2.5_ over long periods may pose a greater risk to human health than originally assumed.

